# The impact of internet use on health investment behavior among middle-aged and elderly people: evidence from China

**DOI:** 10.3389/fpsyg.2025.1612115

**Published:** 2025-07-14

**Authors:** Xin Yang, Mengjie Bie, Yuzheng Zhang, Yundong Li

**Affiliations:** School of Ethnology and Sociology, Yunnan University, Kunming, China

**Keywords:** the internet, health investment, middle-aged and elderly people, health information capital, propensity score matching

## Abstract

**Background:**

With the continuous deepening of global aging, improving the health level of the elderly has become one of the most critical issues. Health investment behavior is the key for middle-aged and elderly people (over 45 years old) to prevent future health risks. This study aims to explore the relationship between Internet use and health investment behavior among middle-aged and elderly people.

**Methods:**

A hypothesis model was constructed to explore the impact of internet usage on the health investment behavior of middle-aged and elderly people. The research was based on the data of 17,813 middle-aged and elderly people aged 45 and above from the 2018 China Health and Retirement Longitudinal Study. Quantitative analysis and fixed-effect models were employed to investigate the relationship between internet usage and the health investment behavior of middle-aged and elderly people.

**Results:**

The use of the Internet has a significant positive impact on the health investment behavior of middle-aged and elderly people, specifically manifested as a 4.2% increase in the probability of health investment and a 29.8% increase in the scale of health investment. This effect is statistically significant at the 1% level. The accumulation of health information capital plays a mediating role in the relationship between Internet use and the health investment behavior of middle-aged and elderly people, while educational attainment plays a moderating role in this process. In addition, the impact of Internet use on the health investment behavior of middle-aged and elderly people is heterogeneous, and this difference is mainly reflected in factors such as place of residence, income level, and age. Overall, Internet use has a more significant impact on the health investment behavior of urban residents, those with better economic conditions, and younger groups.

**Conclusion:**

The positive impact of Internet usage on the health investment behavior of middle-aged and elderly people is of great significance. The Internet can provide more opportunities for middle-aged and elderly people to obtain health information. The more they seek health information capital, the more obvious the effect of promoting health investment behavior will be.

## 1 Introduction

With the continuous extension of individual life expectancy, population aging has become a global social issue. To effectively improve the health level and quality of life of the elderly, the World Health Organization (WHO) has released the “World Report on Ageing and Health” and formulated the Decade of Healthy Ageing 2020–2030 Action Plan (Rudnicka et al., 2020). This move indicates that the concern of the whole society for the health of the elderly has reached a new height. Health investment refers to the time cost and medical expenses invested to maintain the current health status and prevent potential diseases (Kim et al., 2023). This behavior shifts health expenditure or health consumption from a “passive” mode to an “active” mode, with preventive health investment as the main focus (Yaoling and Yangyang, 2023). With the advent of the digital economy era, the “digital dividend” brought about by the rapid development of the Internet has had a profound impact on the health investment behavior of middle-aged and elderly people. According to the data released by the China Internet Society (CNNIC), as of 2024, the elderly population aged 60 and above in China has reached 310 million, accounting for 22.0% of the total national population, among which the population aged 65 and above is 220 million, accounting for 15.6% of the national population. This data indicates that the penetration rate of the Internet among the elderly continues to rise (Li et al., 2025). Currently, the Internet has become an important channel for middle-aged and elderly people to obtain health information and manage their health (Hunsaker et al., 2021). Meanwhile, the frequent occurrence of problems such as chronic diseases and the uneven distribution of medical resources has prompted middle-aged and elderly people to pay more attention to health management (Chun et al., 2024). The inherent convenience and efficiency of the Internet have lowered the threshold for middle-aged and elderly people to obtain health information and provided an effective way for them to invest in their health.

The impact of Internet usage on population health and health management behaviors has drawn the attention of scholars (Guo et al., 2022). Internet technology and the field of health care continue to deepen and integrate, affecting the population’s health investment decisions and the realization of the form (Nie et al., 2023). Relevant studies have shown that the development of the Internet has greatly improved the health of the population and suppressed the mortality rate (Wu et al., 2022; Du et al., 2024), Internet use affects the physical health of individuals through health literacy mechanisms (Luo et al., 2024). In addition, seniors who use the Internet have higher impacts on self-rated health, mediated by social support from loved ones and friends (Liu et al., 2022). There is also a correlation between regional Internet development and individual mental health, with provinces with higher levels of Internet development having fewer symptoms of depression among middle-aged and older adults (Zhong and Wang, 2023; Zhou and Ding, 2023), but excessive Internet use can also have a certain impact on their mental health (Visnjic et al., 2024). Research indicates that residents’ health investment behaviors are influenced by multiple factors, including technological development, economic level, cultural background, and life cycle stage (Yoon et al., 2020). Additionally, an individual’s mental health status significantly promotes health-related expenditures, a phenomenon that is particularly prominent in low-income families (Shuyong et al., 2024). Meanwhile, existing studies have confirmed that there is a correlation between healthcare investment and social economic growth. Health investment can effectively promote economic growth, but its impact effect shows a nonlinear growth characteristic (Wang et al., 2025).

It is worth noting that the current research mainly focuses on the relationship between Internet usage and health. However, as an important component of healthy behaviors, health investment plays a key role in enhancing the health capital of middle-aged and elderly people (aged over 45). Currently, research on the impact of Internet usage on the health investment of middle-aged and elderly people and its potential mechanisms is still insufficient, and it fails to take middle-aged and elderly people with higher health needs as the research objects more specifically. An in-depth exploration of the influence of the Internet on their health investment behavior and its mechanism of action is of great practical significance for improving the healthy lifestyle and enhancing the health level of this group. Therefore, from the perspective of preventive health investment, this study utilizes the relevant data from the China Longitudinal Study on Health and Retirement (CHARLS) to evaluate the impact of Internet usage on health investment among middle-aged and elderly people. This research is of great significance for filling the existing research gaps and deepening the understanding of related fields.

## 2 Theoretical frameworks

Dr. Grossman proposed the health demand theory in 1972, which regards health as capital stock, and points out that individual health capital stock can be influenced through health investment behavior, thereby improving their health status (Grossman, 1972). An individual’s health behaviors mainly include health prevention behaviors, basic health behaviors, protective behaviors, and health service utilization behaviors (Tang and Wang, 2023). Among them, health investment, as the core content of health prevention behavior, involves the time and expenses that individuals invest to maintain their current health status or prevent potential diseases, including related expenditures such as preventive physical examinations, vaccinations, fitness exercises or the purchase of healthy food (Abubakar et al., 2022; Hunsaker and Hargittai, 2018).

With the rapid development of the Internet, the scope and efficiency of health information dissemination have been significantly enhanced. For the middle-aged and elderly population at specific life stages, the Internet offers them opportunities to learn about disease prevention, scientific medication use, and other related knowledge, effectively reducing health cognition misunderstandings and simultaneously improving their accessibility to health information and their awareness of their health (Han and Zhao, 2021). Against this backdrop, the concept of “preventive health investment” has gradually deepened among the middle-aged and elderly, further increasing the probability of them engaging in self-health management and investment.

Furthermore, the rapid development of information technology has promoted the wide application of online payment. To accumulate health capital to maintain or improve their health conditions, convenient online consumption and payment methods provide strong support for middle-aged and elderly people to expand the scale of health investment. Therefore, we believe that the popularization of Internet usage has a significant positive effect on promoting the probability and scale of health investment among middle-aged and elderly people. Based on the above analysis, this paper proposes the following research hypothesis:

*H1*: Internet usage can positively influence the probability and scale of health investment among middle-aged and elderly people.

Internet use can improve access to health information through low-cost health information delivery and sharing for middle-aged and older adults, who may use the Internet to search, browse, evaluate, select, and use health information to meet their personal health information needs, which in turn affects their health investments (Peng and Chan, 2020). Compared with taking offline health consultation, the Internet provides residents with health information through precise positioning with the characteristics of interactivity and relevance, in addition to saving the time and cost costs of consulting with offline medical institutions, middle-aged and old people are constrained by the flexibility of physical functions, they are more willing to use the Internet to obtain health information. The Internet provides a rich resource environment for residents to obtain health information, which is conducive to middle-aged and old-aged people’s ability to enhance their ability to obtain and screen health information, but overly abundant health information can also lead to users’ triggering of health anxiety (Byaro et al., 2023). In conclusion, middle-aged and elderly people can accumulate online health-related knowledge through the use of the Internet. The acquisition of health information capital can help them make informed health decisions. By providing targeted health information, this information has had a positive impact on the health knowledge base of middle-aged and elderly people, helping to reduce their personal health risks and thereby increasing their investment in health to enhance health capital (Yifeng, 2024). In addition, as middle-aged and older people age and their physical functions gradually decline, they need to spend more time and energy using new digital skills, and many older people are also fearful of the use of digital technology and have difficulties in learning and using the Internet (Chen et al., 2024). In contrast, well-educated middle-aged and elderly people have a deeper understanding of digital information technology. They themselves have better quality education and knowledge and skills, and can better utilize the Internet to obtain health information and meet their personal health service needs (Shi and Zhang, 2023). Based on this, the research hypothesis is formulated:

*H2*: Access to information capital plays a mediating role in moderating the positive impact of Internet use on health investment generation among middle-aged and older adults.

*H3*: Educational attainment plays a positive role in moderating the impact of Internet use on the health investment generated by middle-aged and older adults.

The impact of Internet usage on health investment among middle-aged and elderly people may exhibit heterogeneity due to individual characteristic differences. Specifically, because of the different characteristics of the places where middle-aged and elderly people live, the significant differences in the economic development levels of urban and rural areas lead to notable distinctions in the completeness of communication infrastructure construction, which may further affect the behavior and effectiveness of middle-aged and elderly people in rural and urban areas in making health investments through the Internet (Zhao and Guo, 2020). Income disparity among individuals may have heterogeneous effects on the attitudes of middle-aged and elderly people toward health investment after they use the Internet. Specifically, middle-aged and elderly groups with a certain economic foundation tend to have a broader range of health investment when making health investment decisions than those with lower incomes.

In addition, the differences in individual age characteristics have a significant impact on Internet usage and its application depth, which may in turn have varying degrees of influence on the health investment behaviors of different age groups (Cotten et al., 2014). Specifically, younger groups tend to maintain a positive and optimistic attitude after obtaining health information and actively make health investment decisions; while older groups, due to the irreversible decline in physical functions (Oswald and Wagner, 2023), usually show a more negative attitude and thus take fewer health investment actions. Based on the above analysis, this study proposes the following hypothesis:

*H4*: The impact effect of Internet usage on the health investment of middle-aged and elderly people varies significantly due to income level, type of residence, and age characteristics.

**Figure 1:**
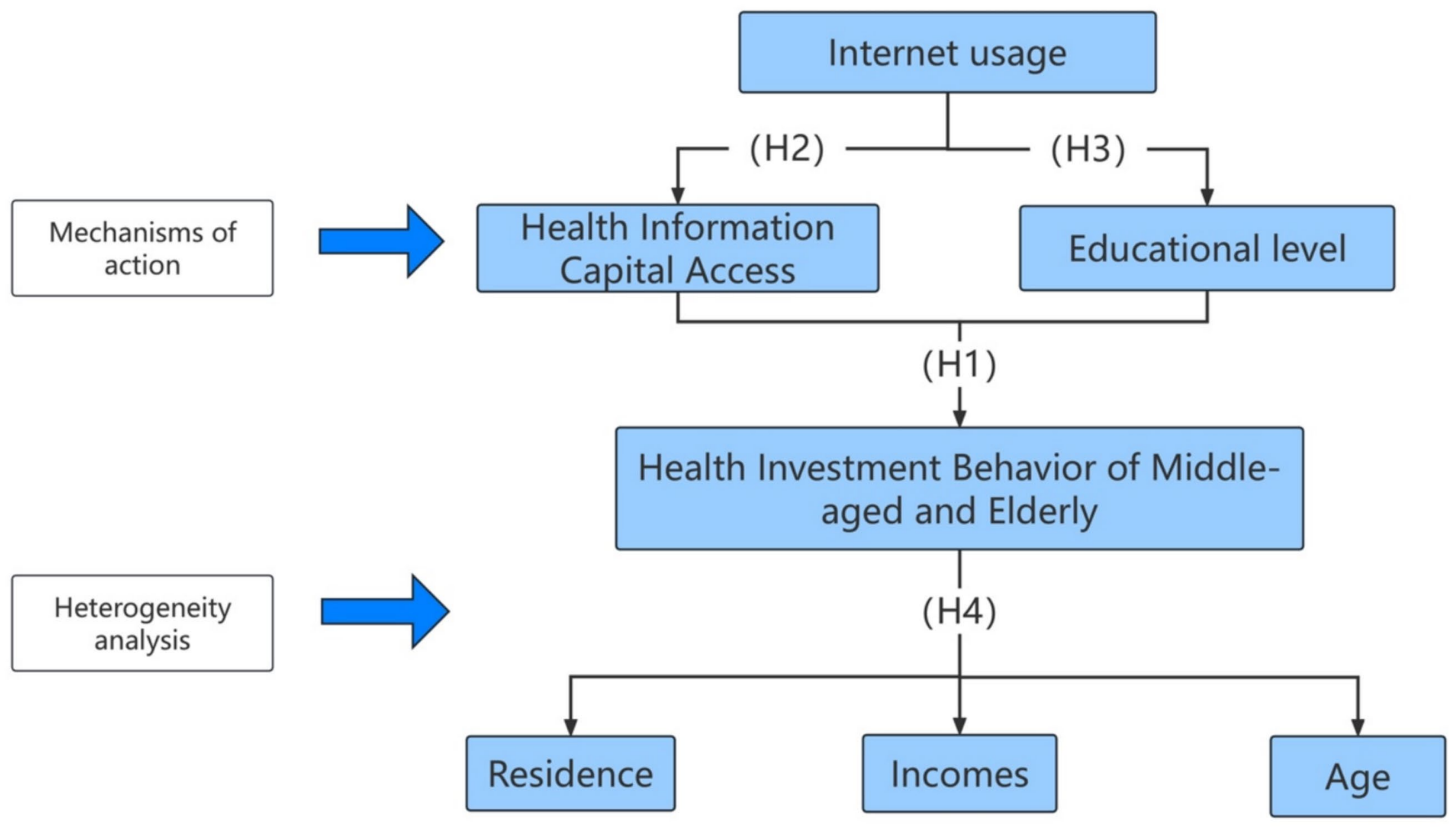
Structure of the proposed theoretical analysis model.

Figure 1 presents the theoretical hypothesis model of the relationship among Internet usage, acquisition of information capital, and health investment behavior constructed based on the above analysis.

## 3 Data and methods

### 3.1 Data source and sample

The study population was middle-aged and older adults (age>45), and we chose data from the China Health and Retirement Longitudinal Study (CHARLS) 2018 as the empirical sample. The database uses middle-aged and elderly people aged 45 and above in 150 districts and counties in China as survey respondents, and the research includes issues related to the study of population aging. The database began a national tracking survey in 2011 and is currently completing a national tracking survey by 2020, with 19,000 questionnaire respondents. The questionnaire includes a wealth of questions related to the sample’s family, personal information, physical functioning, lifestyle, health care, and other relevant questions for scholars to conduct relevant research. The database is widely used in China in the fields of sociology and public health at the micro level of old age for scholars to conduct related research. CHARLS was approved by the Ethics Review Board of Peking University and the survey was informed by a written statement of knowledge provided by the interviewer (Yu and Fiebig, 2020). Samples with age ranges greater than 45 and above were retained, and after data cleaning, 17,813 observations with complete information on features were finally obtained.

### 3.2 Variable selection

#### 3.2.1 Dependent variable

The dependent variable of this study is the health investment of middle-aged and elderly people. To accurately express the impact of Internet use on the health investment behavior of middle-aged and elderly people, following the practices of existing scholars (Shi and Zhang, 2023), the explained variable is mainly constructed through two variables: the probability and scale of health investment. The probability of health investment for middle-aged and elderly people is determined by the question in the questionnaire: “Did you spend on health care in the past year?” To measure it, 1 indicates that health care expenditure has been made, and 0 indicates no. The scale of health investment is indicated by “the amount of health care expenditure in the past year.” The scope of health care expenditure includes preventive care, fitness exercises and products and equipment, health supplements, etc., and the corresponding values are taken. When middle-aged and elderly people use their income for health investment, it can effectively reduce health risks, improve personal health human capital, and enhance health levels (Halliday et al., 2019).

#### 3.2.2 Independent variables

The independent variable of this study is the internet usage of middle-aged and elderly people. Based on the responses to the original questionnaire, the question “Have you engaged in the following social activities in the past month?” is used as the measurement indicator for internet usage among middle-aged and elderly people. If the sample’s response indicates that they used the internet to go online, the sample is assigned a value of 1; otherwise, it is assigned a value of 0.

#### 3.2.3 Control variables

This study mainly selects sociodemographic characteristics, family characteristics, and individual health characteristics as control variables. The sociodemographic characteristics level includes: gender, age, ethnicity, marital status, educational attainment, and place of residence; the family characteristics level includes average family income and family assets; the individual health characteristics level includes self-rated health, chronic diseases, health accidents, and social medical insurance (individual participation status).

### 3.3 Methods

First, to explore the impact of Internet use on the health investment of middle-aged and elderly people, a two-way fixed-effects model was used for the assessment. The constructed regression model is as shown in Equation 1:


(1) $$H_{it}=\alpha_{0}+\beta_{0}\delta_{\mathrm{it}}+\gamma {Τ}_{\mathrm{it}}+{Ζ}_{t}+\omega_{i}+\mu_{\mathrm{it}}$$

$\;H_{it}$ represents the health investment of the i variable after Internet use, including the probability and scale of health investment among the middle-aged and elderly. $\delta_{\mathrm{it}}$ and ${Τ}_{\mathrm{it}}$ indicated the Internet use and individual characteristics of middle-aged and elderly people; ${Ζ}_{t}$ and $\omega_{i}$ are individual and household fixed effects; $\beta_{0}$ and γ were regression coefficients, respectively. $\mu_{\mathrm{it}}$ is a random disturbance term.

Secondly, to reduce the interference of endogeneity and errors on the robustness of the results and enhance the robustness of the test results, we used Propensity score matching (PSM) methods for a second regression, with the model as follows:


(2) $$P_{i}(x)=P_{j}\;(D_{i}=1\backslash x_{i})=\text{Logit}[f(x_{i})]$$

Equation 2 represents the linear function of the covariates of the sample. Assign “treatment group = 1” to middle-aged and elderly people who have used the Internet, and “control group = 0” to other samples. To calculate the sample propensity score, all the factors affecting the health investment of middle-aged and elderly people should be incorporated into the model, and the propensity score of the covariate should be calculated by regression. Secondly, the treatment group and the control group should be matched according to the calculated score, so that the two groups of samples are the same in terms of characteristics. Finally, a set of sample data that can effectively overcome the endogeneity problem is obtained by eliminating the samples that do not meet the requirements.

## 4 Results

### 4.1 Descriptive analysis

Table 1 summarizes the basic characteristics and descriptive statistics of each variable. The sample includes 17,813 middle-aged and elderly individuals. In this example, the probability and scale of health investment among middle-aged and elderly people are 0.081 ± 0.273 and 0.485 ± 1.849 respectively, and the average value of the sample using the Internet is 0.131 ± 0.338. The results show that the proportion of middle-aged and elderly people using the Internet and having health investment behavior is relatively small, and the correlation between the two needs further exploration. From the control variables, it can be seen that the average age of the middle-aged and elderly population is about 61.99 years old, and the sample mainly consists of female middle-aged and elderly people, with the majority living in rural areas and a relatively small number suffering from chronic diseases.

**Table 1:** Descriptive statistics results of variables (mean ± SD).

Variable	Variable definition	Obs	Total	*p-*value
Probability of investment in health	0 = No 1 = Yes	17,813	0.081 ± 0.273	<0.01***
The scale of investment in health	Continuous variable	17,813	0.485 ± 1.849	<0.001***
Internet usage	0 = No 1 = Yes	17,813	0.131 ± 0.338	<0.01***
Gender	0 = Female 1 = Male	17,813	0.475 ± 0.499	<0.05*
Age	Continuous variable	17,813	61.996 ± 10.116	<0.001***
Nationality	0 = Other 1 = Han Chinese nationality	17,813	0.920 ± 0.271	<0.001***
Marital status	0 = Otherwise 1 = Coupled,	17,813	0.848 ± 0.358	<0.001**
Educational level	Continuous variables	17,813	3.444 ± 1.940	<0.01**
Residence	0 = Urban 1 = Rural	17,813	0.711 ± 0.453	<0.001***
Income level	Continuous variable	17,813	2.331 ± 4.172	<0.001**
Family assets	Continuous variable	17,813	0.480 ± 2.245	<0.01**
Self-assessed health	Continuous variable, Values 1–5: very bad; bad; fair; good; very good	17,813	3.049 ± 1.024	<0.001***
Chronic health condition	0 = No 1 = Yes	17,813	0.446 ± 0.497	<0.01***
Health accident	0 = No 1 = Yes	17,813	0.001 ± 0.035	<0.01**
Social health insurance	0 = No 1 = Yes	17,813	0.143 ± 0.350	<0.001**

*p < 0.1, **p < 0.05, ***p < 0.01.

### 4.2 Baseline regression

Table 2 shows the results obtained from the regression based on the two-way fixed effects model, indicating the effect of Internet use on the health investment behavior of middle-aged and elderly people.

**Table 2:** Baseline regression results.

Variable	Probability of investment in health			The scale of investment in health		
	(1)	(2)	(3)	(4)	(5)	(6)
Internet usage	0.090*** (0.005)	0.052*** (0.006)	0.042*** (0.006)	0.355***(0.097)	0.394*** (0.042)	0.298*** (0.043)
Gender		−0.020*** (0.004)	−0.019*** (0.004)		−0.162*** (0.028)	−0.153*** (0.029)
Age		0.001*** (0.0002)	0.001*** (0.0002)		0.015*** (0.001)	0.011*** (0.001)
Nationality		−0.027*** (0.007)	−0.028*** (0.007)		−0.207*** (0.048)	−0.199*** (0.050)
Marital status		−0.0008 (0.005)	0.002 (0.005)		0.064 (0.039)	0.066 (0.041)
Educational level		0.013*** (0.001)	0.009*** (0.001)		0.109*** (0.008)	0.075*** (0.008)
Residence		−0.074*** (0.004)	−0.052*** (0.005)		−0.551*** (0.031)	−0.391*** (0.034)
Income level		0.0006 (0.0005)	0.0004 (0.0005)		0.006* (0.003)	0.002 (0.003)
Family assets		0.0001 (0.0008)	0.0009 (0.0008)		0.010 (0.005)	0.015*** (0.006)
Self-assessed health			0.002 (0.002)			0.013 (0.013)
Chronic health condition			0.010*** (0.004)			0.075*** (0.028)
Health accident			0.073 (0.060)			0.123 (0.423)
Social health insurance			0.077*** (0.006)			0.636*** (0.045)
Household fixed effect	Yes	Yes	Yes	Yes	Yes	Yes
Personal fixed effect	Yes	Yes	Yes	Yes	Yes	Yes
Constant	−2.710*** (0.029)	−0.006 (0.018)	−0.004 (0.020)	7.063*** (0.052)	−0.292** (0.127)	−0.196 (0.142)
*N*	17,813	17,813	17,813	17,813	17,813	17,813
*R* ^2^	0.0175	0.0448	0.0518	0.0091	0.0553	0.0647

*p < 0.1, **p < 0.05, ***p < 0.01. Clustered standard errors were in brackets.

As shown in Table 2, columns (1) through (3) represent the regression results of sequentially adding different levels of control variables to the probability of health investment in middle-aged and older adults, controlling for household and individual fixed effects. As can be seen from the regression results obtained in Column (3) with the inclusion of individual social, family, and health characteristics, the estimated coefficient of Internet use is 4.2% (*p* < 0.01), which has a significant effect on the probability of investing in the health of middle-aged and elderly people. Columns (4) to (6) represent the results of separate regressions of health investment size after adding control variables sequentially, and column (6) shows the regression results after adding all the sample characteristics; Internet use still has a positive impact on the health investment size of middle-aged and elderly people, with an estimated coefficient of 29.8% (*p* < 0.01). H1 is validated, and to ensure the robustness of the regression results, the benchmark regression results are again evaluated in this study.

### 4.3 Robustness test

The benchmark regression analysis results of this paper show that the use of the Internet has a significant impact on the probability and scale of health investment among the middle-aged and elderly population. However, there are potential endogeneity problems and unobserved omission of variables in the model setting, which may lead to deviations in the regression results. Therefore, in order to effectively solve the problems of endogeneity and sample self-selection, this study adopted the two-stage instrumental variable method, the PSM method and the replacement of independent variables to conduct robustness tests.

#### 4.3.1 Instrumental variable method

Drawing on the practices of existing scholars (Jiali and Chenlei, 2024), the broadband penetration rate of a certain region in the previous year was selected as the instrumental variable for the Internet usage of middle-aged and elderly people. The reason for choosing this instrumental variable is that the internet usage of middle-aged and elderly people may be influenced by factors such as personal income, regional development, and educational level, inevitably leading to endogeneity issues. Therefore, the broadband penetration rate of the region where the respondents are located in the previous year is selected as an instrumental variable for two-stage estimation to effectively address the endogeneity problem of internet usage among middle-aged and elderly people. There is a close connection between the broadband penetration rate of a region and the internet usage of its residents. A higher broadband penetration rate in the previous year implies a higher internet penetration rate in the current period, and a greater probability of middle-aged and elderly people using the internet, satisfying the requirement of relevance for the instrumental variable. However, the broadband penetration rate of the previous year has no direct and inevitable connection with the health investment behavior of middle-aged and elderly people in the current period. It can influence the health investment behavior of middle-aged and elderly people by affecting their internet usage in the current period, meeting the exogeneity principle for the use of instrumental variables.

The regression results are presented in Table 3. In the first stage of the regression analysis, the instrumental variable is confirmed to be positive at the 1% significance level, indicating a significant correlation between the instrumental variable and the internet usage of middle-aged and elderly people. The *p*-value of the Kleibergen-Paap rk LM statistic is 0.0000, showing extremely high significance. The value of the Cragg-Donald Wald *F* statistic for the weak instrumental variable test is 19.525, which is much higher than the critical value of 16.380 at the 10% level of Stock-Yogo, indicating that there are no weak identification or non-identification problems with the instrumental variable. The results show that after introducing the instrumental variable to consider endogeneity, the internet usage in the second stage of 2SLS has a significant impact on the probability and scale of health investment by middle-aged and elderly people, indicating the robustness of the benchmark regression results.

**Table 3:** Estimation results of instrumental variables.

Variable	The first stage	The second stage	
		Probability of investment in health	The scale of investment in health
Internet usage		1.464*** (0.388)	8.554*** (2.344)
instrumental variable	0.008*** (0.002)		
Control variable	Yes	Yes	Yes
Kleibergen-Paap rk LM		18.208 [0.0000]	
Cragg-Donald Wald F		19.525 {16.380}	
N	17,813	17,813	17,813

The Kleibergen-Paap rk LM statistic is used for the unidentifiable test, and the *p* value is within []. The Cragg-Donald Wald *F* statistic is used for the weak instrumental variable test, where {} represents the critical values of the Stock-Yogo weak ID test critical values at the 10% level. *p < 0.1, **p < 0.05, ***p < 0.01. Clustered standard errors were in brackets.

#### 4.3.2 PSM method

**Figure 2:**
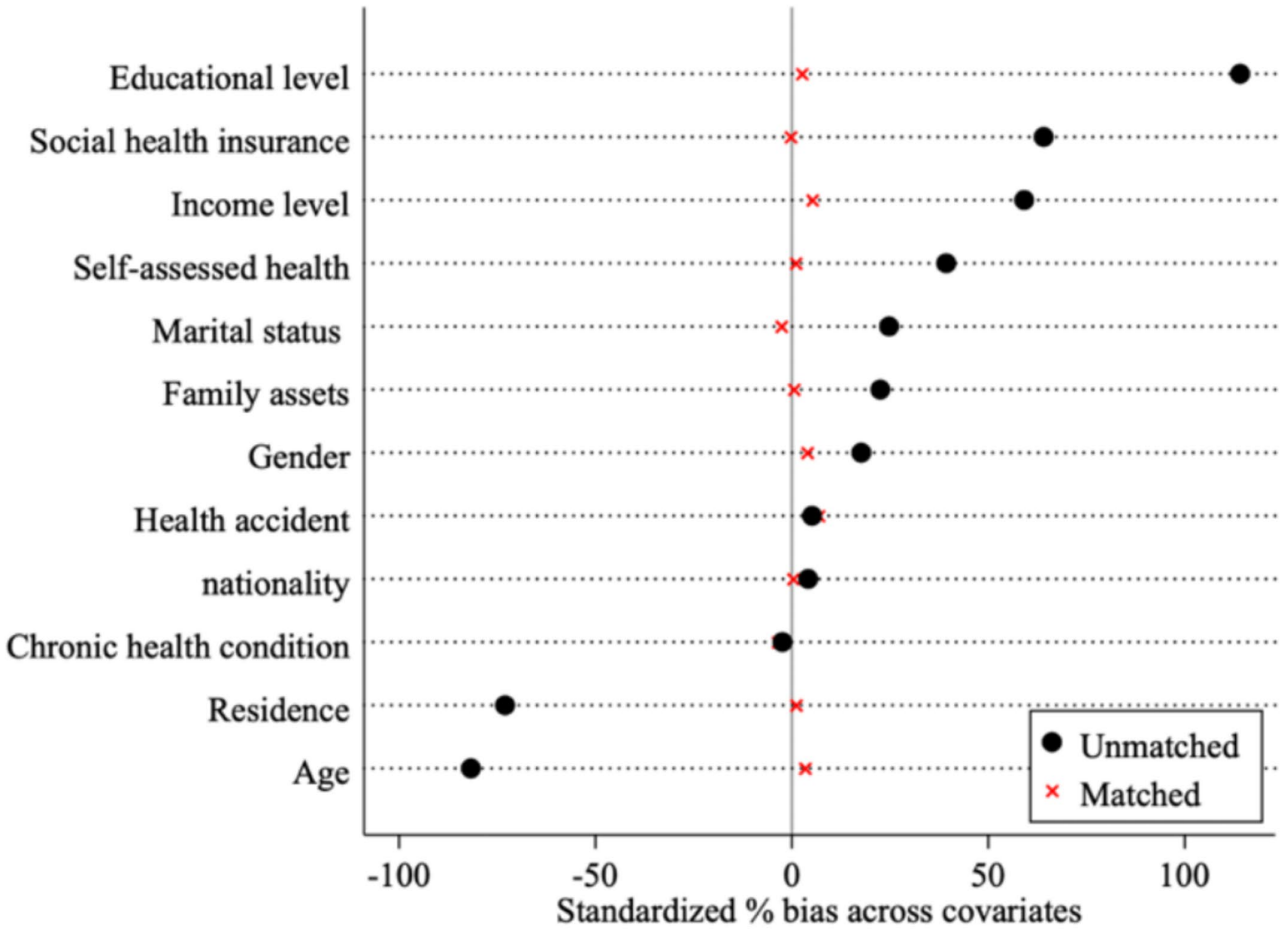
Comparison chart of the standard deviation of covariates before and after PSM matching.

Due to the differences in the characteristics of the assessment samples, the problem of sample self-selection easily exists. To further overcome the sample endogeneity problem, a “counterfactual sample” was constructed using the k-nearest-neighbor matching method (1:1) to test the “net effect” of policy implementation. Figure 2 shows the comparison before and after covariate matching, with the standard deviation gradually converging to 0, further reducing the sample self-selection problem. Table 4 lists the results after matching through the PSM model (K-nearest neighbor matching and Mahalanobis distance matching), which are consistent with the baseline regression results and verify Hypothesis H1.

**Table 4:** Estimation results of the PSM method.

Variable	Probability of investment in health		The scale of investment in health	
	K-nearest Neighbor Matching (1:1)	Mahalanobis Distance Matching	K-nearest Neighbor Matching (1:1)	Mahalanobis Distance Matching
Internet usage	0.041*** (0.006)	0.047*** (0.010)	0.300*** (0.044)	0.296*** (0.070)
Control variable	Yes	Yes	Yes	Yes
Constant	−0.0007 (0.020)	−0.035* (0.047)	−0.208 (0.146)	−0.439*** (0.326)
*N*	17,375	4,655	17,375	4,655
*R* ^2^	0.0527	0.0528	0.0650	0.0623

*p < 0.1, **p < 0.05, ***p < 0.01. Clustered standard errors were in brackets.

#### 4.3.3 Replace the independent variable

The health investment behavior of middle-aged and elderly people may be affected by factors such as personal health and economic status. To avoid the influence of reverse causality on the estimated results of this study, we conducted the verification again by replacing the independent variables. The variable of whether to use the Internet was replaced with the individual Internet usage frequency, and control variables at the individual and family levels were added. The regression results after replacing the independent variables are shown in Table 5. It can be seen that the use of the Internet has had a significant positive impact on the probability and scale of health investment in middle-aged and elderly people, and the research hypothesis H1 has been verified.

**Table 5:** Replace the estimation result of the independent variable.

Variable	Probability of investment in health	The scale of investment in health
Internet usage	0.016*** (0.002)	0.117*** (0.015)
Control variable	Yes	Yes
Constant	−0.004** (0.020)	−0.198** (0.142)
*N*	17,813	17,813
*R* ^2^	0.0524	0.0653

*p < 0.1, **p < 0.05, ***p < 0.01.

### 4.4 Intermediary mechanisms and moderating effects

Based on the above analysis and drawing on the viewpoints of existing scholars (Cui et al., 2024), the mechanism by which internet usage affects the health investment behavior of middle-aged and elderly people may be through enhancing the accumulation of health information capital among this group, thereby influencing their degree of emphasis on health and subsequently their health investment. Columns (1) to (3) of Table 6 present the regression results of health information capital acquisition as a mediating variable for the impact of internet usage on the health investment of middle-aged and elderly people. The results obtained indicate that this influence mechanism has a significant effect, suggesting that health information capital acquisition plays a mediating role in the impact of internet usage on health investment.

**Table 6:** Regression results for mediating mechanisms and moderating effects.

Variable	Health Information Capital Access	Probability of investment in health	The scale of investment in health	Educational level	Probability of investment in health	The scale of investment in health
	(1)	(2)	(3)	(4)	(5)	(6)
Internet usage	0.016*** (0.002)	0.043*** (0.015)	0.299*** (0.108)	0.009*** (0.006)		
Health information capital access		−0.001 (0.016)	−0.001 (0.113)			
Internet usage *Educational level					0.019*** (0.003)	0.124*** (0.023)
Control variable	Yes	Yes	Yes	Yes	Yes	Yes
*N*	17,813	17,813	17,813	17,813	17,813	17,813

To further analyze the role of educational attainment among middle-aged and elderly individuals in the process of how internet usage affects health investment behavior, we take the interaction term of internet usage and educational attainment as a moderating variable. The results are presented in columns (4) to (6) of Table 6. The results of the moderating effect show that the interaction effect of internet usage and educational attainment has a positive impact on both the probability and scale of health investment, with estimated coefficients of 1.9% (*p* < 0.01) and 12.4% (p < 0.01), respectively. This indicates that educational attainment plays a moderating role in the mechanism by which internet usage affects health investment behavior, and middle-aged and elderly individuals with higher educational attainment are more likely to engage in health investment behavior through internet usage. Therefore, H2 and H3 are verified.

### 4.5 Heterogeneity analysis

The popularization of the Internet has indeed had a significant impact on the health investment behavior of middle-aged and elderly people. However, due to the characteristics of Internet technology itself (such as information overload, operational complexity, and virtuality) as well as the heterogeneity of the middle-aged and elderly population (age, education level, economic status, etc.), this impact shows obvious differences. This study examines the heterogeneity of the impact of Internet use on the health investment behavior of middle-aged and elderly people from different dimensions, and the results are shown in Table 7.

**Table 7:** Heterogeneity analysis results.

Variable	(1) Rural	(2) Urban	(3) Higher incomes	(4) Lower incomes	(5) Age<60	(6) Age:60–79	(7) Age> = 80
Probability of investment in health							
Internet usage	0.032*** (0.007)	0.048*** (0.012)	0.050*** (0.010)	0.036*** (0.008)	0.037*** (0.007)	0.048*** (0.012)	0.004 (0.072)
Control variable	Yes	Yes	Yes	Yes	Yes	Yes	Yes
Constant	0.002 (0.019)	−0.144*** (0.052)	−0.033 (0.047)	0.017 (0.022)	0.020 (0.019)	0.143*** (0.016)	0.111 (0.050)
*N*	12,776	5,037	4,386	13,427	7,921	8,542	894
The scale of investment in health							
Internet usage	0.227*** (0.045)	0.346*** (0.092)	0.320*** (0.073)	0.289*** (0.055)	0.243*** (0.051)	0.424*** (0.086)	0.113 (0.473)
Control variable	Yes	Yes	Yes	Yes	Yes	Yes	Yes
Constant	0.002 (0.019)	−0.144*** (0.052)	−0.033 (0.047)	0.017 (0.022)	0.020 (0.019)	0.143*** (0.016)	0.111 (0.050)
*N*	12,776	5,037	4,386	13,427	7,921	8,542	894

Table 7, columns (1) to (7), explores the heterogeneous impact of internet usage on the health investment behavior of middle-aged and elderly people in different residential areas, economic conditions, and age groups. The results show that the impact of internet usage on the health investment behavior of middle-aged and elderly people in urban areas is greater than that in rural areas; middle-aged and elderly people with higher incomes are more likely to make health investment decisions and have a larger scale of investment after using the internet compared to those with lower incomes; the impact of internet usage on the health investment of middle-aged and elderly people aged 60 to 79 is more significant, but there is no significance for those over 80 years old. Therefore, H4 is verified.

## 5 Discussion

With the continuous intensification of population aging, the health issues of middle-aged and elderly people have gradually become a social topic of global concern. Preventive health investment behavior plays a crucial role in improving the health status of middle-aged and elderly people. Previous studies have mostly focused on exploring the impact of Internet use on the overall health of residents. However, this study, based on existing research results, further analyzes the specific impact of Internet use on the prevention of future health risks and health investment behavior of middle-aged and elderly people (Zhao et al., 2016; Soroya et al., 2021). This study is based on the CHARLS database and uses a two-way fixed effect model for regression analysis. The endogeneity and model robustness are tested through the instrumental variable method and propensity score matching method (PSM). In addition, this study also deeply explores the mediating mechanism and moderating effect of the impact of Internet use and conducts heterogeneity impact analysis for different groups. The above research findings provide an important theoretical basis and practical guidance for improving the health level of middle-aged and elderly people and are conducive to optimizing health investment strategies.

This study finds that the use of the Internet has a significant positive impact on the probability and scale of health investment among middle-aged and elderly people. This result indicates that the use of the Internet has enhanced the efficiency of middle-aged and elderly people in obtaining health knowledge and disease prevention information. Through health-related websites or social media platforms, middle-aged and elderly people can quickly access relevant health information, thereby effectively enhancing their self-health management capabilities (Zhao et al., 2025; Urrehman, 2022). In addition, the convenience and information transparency of the Internet enables middle-aged and elderly people to more easily compare prices and thus choose more suitable health investment methods for themselves. Meanwhile, discussions on healthy aging on the Internet may further enhance the awareness of middle-aged and elderly people regarding health risks and raise their consciousness of managing their own health investments.

In terms of the mediating mechanism and moderating effect, the use of the Internet has an impact on the health investment behavior of middle-aged and elderly people through the accumulation of health information capital, which is consistent with the findings of previous scholars (Zhang et al., 2022). One possible reason is that middle-aged and elderly people have reduced the cost of information collection and acquisition through the Internet. By coming into contact with health science popularization content on wechat official accounts, such as short videos and health science popularization articles, as well as obtaining health information through mobile applications, social media or websites and other channels, they have realized the harm of bad living habits and applied the health information they have obtained to health investment, thereby increasing their health capital (Liang et al., 2022). Furthermore, middle-aged and elderly people with a higher level of education can utilize online health information more effectively than individuals with a lower level of education and are more inclined to make large-scale health investments via the Internet. This might be because the information literacy of highly educated groups is relatively high, enabling them to effectively distinguish the authenticity of online health information and make scientific health investments through professional platforms (Neely et al., 2022).

In terms of heterogeneity, the research found that after using the Internet, urban residents, those with better economic conditions, and younger groups are more likely to make health investments than rural residents, those with lower incomes, and those over 80 years old. The possible reasons are as follows: Firstly, due to the development gap between urban and rural areas, the degree of network coverage varies among different regions. The penetration rate of smartphones among rural elderly people is low, and there is a significant digital divide, resulting in a lower utilization rate of the Internet. Secondly, elderly people with better economic conditions have more ability to convert Internet information into actual health investments. Finally, younger groups are more familiar with the application and operation of the Internet, and can actively obtain health information through social media or health apps, etc., so they are more proactive in health investment behavior than the elderly (Lee and Jang, 2022).

Based on the conclusions of this study, the following policy recommendations are put forward: First, to enhance the health investment level of the middle-aged and elderly groups and ensure that rural elderly people can fully enjoy the health dividends brought by the development of the Internet, efforts should continue to be made to promote the construction of digital infrastructure in rural areas, increase the Internet penetration rate, and further address the issue of the digital divide in rural areas. Second, to enhance the health management awareness of the middle-aged and elderly groups, it is necessary to tailor health education programs for low-income middle-aged and elderly groups, improve the digital literacy of the elderly, and ensure that low-income groups have the opportunity to obtain health investment dividends through the Internet. Thirdly, decision-makers should formulate precise Internet promotion policies for rural areas, low-income groups and the elderly, and implement “precise assistance” policies. Community organizations should regularly conduct popular science training on Internet literacy, strengthen the prevention and regulation of online behavior, and minimize the risks of the elderly making health investments through the Internet as much as possible.

However, this study still has some limitations. First, the research only focuses on the probability and scale of health investment among middle-aged and elderly people, and fails to comprehensively explore health investment behavior throughout the entire life cycle and its relationship with family decisions. Second, the indicator system used in the study to measure health investment behavior is relatively limited and does not fully cover all relevant aspects, including vaccination, healthy diet, and exercise behavior. Future research can further explore these areas. Third, when selecting control variables, the study failed to include factors that may affect the health investment of middle-aged and elderly people, such as environmental pollution or environmental policies (Liao et al., 2025; Zheng et al., 2025). Future research can further control for these factors. Fourth, the use of 2018 CHARLS data in this study has limitations in determining causal relationships. It is recommended that future research combine specific dimensions and behaviors not included in this study and use longitudinal data to further determine the causal relationship between internet use and health investment behavior of the group.

## 6 Conclusion

Health investment behavior is an important influencing factor for increasing the health capital stock of the middle-aged and elderly groups. With the advent of the digital society, the use of the Internet has changed the way residents obtain health information and has had an important impact on their health behaviors. This study, based on the 2018 China Health and Retirement Longitudinal Study (CHARLS) database, utilized fixed-effect models, instrumental variable methods, and propensity score matching (PSM) to assess the impact of internet usage on health investment among middle-aged and elderly individuals, and explored the mediating mechanisms and heterogeneous effects. The findings are as follows: First, internet usage has a positive impact on health investment among middle-aged and elderly individuals, increasing the probability of health investment by 4.2% and the investment scale by 29.8%. Second, the accumulation of health information capital plays a mediating role in the influence of internet usage on the health investment behavior of middle-aged and elderly individuals, with educational attainment playing a moderating role in the impact of internet usage. Third, the impact of internet usage on the health investment behavior of middle-aged and elderly individuals varies by place of residence, income, and age, with more significant effects observed among those living in urban areas, those with better economic conditions, and younger individuals within this age group. Based on the concept of “preventive health investment,” this study empirically tested that the use of the Internet has a positive impact on the health investment behavior of middle-aged and elderly people. The conclusion of this study has important practical significance for enhancing the health management awareness of middle-aged and elderly people, improving the healthy lifestyle of this group and enhancing the health level.

## Data Availability

The original contributions presented in the study are included in the article/supplementary material, further inquiries can be directed to the corresponding author.
